# Validity of Apgar Score as an Indicator of Neonatal SARS-CoV-2 Infection: A Scoping Review

**DOI:** 10.3389/fmed.2021.782376

**Published:** 2022-01-11

**Authors:** Melissa Chao, Carlo Menon, Mohamed Elgendi

**Affiliations:** ^1^Faculty of Medicine, University of British Columbia, Vancouver, BC, Canada; ^2^Bloomberg School of Public Health, Johns Hopkins University, Baltimore, MD, United States; ^3^Biomedical and Mobile Health Technology Laboratory, Department of Health Sciences and Technology, Zurich, Switzerland

**Keywords:** COVID-19, well-being, pregnancy, neonates, screening tool, diagnosis, physical examination, score assessment

## Abstract

The coronavirus disease 2019 (COVID-19) pandemic has had profound impacts on healthcare systems worldwide, particularly regarding the care of pregnant women and their neonates. The use of the Apgar score—a discrete numerical index used to evaluate neonatal condition immediately following delivery that has been used ubiquitously as a clinical indicator of neonatal condition and widely reported in the literature for decades—has continued during the pandemic. Although health systems adopted protocols that addressed pregnant women and their neonates during the pandemic, limited research has assessed the validity of Apgar scores for determining neonatal conditions in the context of COVID-19. Therefore, this scoping review was conducted on the first 2 years of the pandemic and included mothers with reverse transcription-polymerase chain reaction confirmed COVID-19 and their resulting positive or negative neonates. In total, 1,966 articles were assessed for eligibility, yielding 246 articles describing 663 neonates. Neonates who tested negative had median Apgar scores of 9 and 9 at 1 and 5 mins, respectively, while test-positive neonates had median Apgar scores of 8 and 9 at the same time points. The proportions of test-negative neonates with Apgar scores below 7 were 29 (4%) and 11 (2%) at 1 and 5 mins, which was not statistically significant (*p* = 0.327, χ^2^ = 0.961). These proportions were even lower for positive neonates: 22 (3%) and 11 (2%) at 1 and 5 mins, respectively, which was not statistically significant (*p* = 1, χ^2^ = 0). The low proportion of Apgar scores below 7 suggests that low Apgar scores are likely to be associated with severe maternal COVID-19 symptoms during delivery rather than neonatal COVID-19. Therefore, this study indicated that Apgar scores are poor indicators of neonatal COVID-19 status.

## Introduction

Beginning in December 2019, the prolific spread of the novel coronavirus known as severe acute respiratory syndrome coronavirus 2 (SARS-CoV-2), which is responsible for coronavirus disease 2019 (COVID-19), resulted in the World Health Organization declaring it a pandemic on March 11, 2020 (1).

As international rates of cases and death tolls continued to rise alarmingly, health systems were forced to adopt new protocols or adapt existing practices to protect vulnerable groups from the virus. One such vulnerable group is neonates, born to mothers with COVID-19, whose developing immune systems may not be equipped to fight off SARS-CoV-2 infection (2). These protocols are necessary in the face of increased complications during pregnancy for women with COVID-19, including increased risks of preterm birth; moreover, one article found that 25% of all neonates born to women with COVID-19 were subsequently admitted to the neonatal care unit, which is above the pre-pandemic rates of admission (3). While data on the impact of COVID-19 on pregnant or recently-pregnant mothers and their neonates continues to emerge, many unanswered questions remain.

One measure of neonatal condition is the Apgar test, a discrete numerical test developed in 1953 by an anesthesiologist at Columbia University, which uses a mnemonic to assess neonatal status, measure response to resuscitation, and compare the outcomes of obstetrical practices, including intrapartum management and effects of maternal pain medication (4). The mnemonic relies on five objective observations: appearance, pulse, grimace, activity, and respiration (5, 6). For a given neonate, each of the five indices is assigned a value from 0 to 2, and the values of each of the five indices are tallied to calculate the total Apgar score (5, 6). Apgar scores above 7 are considered “reassuring,” while scores between 4 and 6 are “below normal,” and scores of 1–3 are “critically low” and typically require urgent clinical intervention (4–6). Due to their ease of application, Apgar scores were ubiquitously implemented to assess neonatal asphyxia without the need for blood gas analyses (4).

Despite the prolific use of Apgar scores in the literature, data from recent reports have suggested a bias in the management of neonates born to mothers with SARS-CoV-2 infection confirmed by reverse transcription polymerase chain reaction (RT-PCR). Recent articles have suggested that the number of neonatal 5 min Apgar scores under 7 during the pandemic has remained comparable to scores from before the pandemic (7, 8). At the same time, various articles have indicated a correlation between high Apgar scores with low complications and low Apgar scores with more frequent or more severe postpartum complications, including cerebral palsy or epilepsy (9–11). In particular, various recent articles have suggested that low Apgar scores are correlated with various complications, including infection by coronaviruses, despite a low proportion of scores below 7 co-occurring with coronavirus infections (12–14).

However, the increasing scrutiny of Apgar scores as a commonly assessed and clinically reported metric in the literature has driven researchers to question the validity of Apgar scores (4, 15, 16). Therefore, this scoping review investigated whether Apgar scores are an accurate indicator of neonatal COVID-19 status and synthesized relevant articles on Apgar scores. It also offers recommendations based on the findings.

## Methods

A review of the literature related to pregnancy and COVID-19 was performed. The study involved five stages: (1) database search, (2) screening according to inclusion and exclusion criteria, (3) review of articles, (4) data analysis, and (5) statistical analysis.

### Database Search

A literature review was conducted for articles published from December 1, 2019 to December 1, 2021. The PubMed and Embase databases were searched using the search terms [(Pregnancy) OR (pregnant woman) OR (pregnant women)] AND [(COVID-19) OR (SARS-CoV-2) OR (coronavirus pregnancy) AND (vertical transmission)].

### Screening According to Inclusion and Exclusion Criteria

Titles and abstracts were screened for eligible articles. The inclusion criteria were (1) written completely in English, (2) outpatient or population-based observational articles (prospective or retrospective), case study reports, or pre-print articles, (3) publication date between December 1, 2019 and December 1, 2021, (4) articles discussing pregnant women diagnosed with COVID-19 using RT-PCR, (5) articles discussing pregnant women in any gestational trimester, (6) articles discussing pregnant women of any maternal age, and (7) articles discuss neonatal outcomes in the context of COVID-19. These included original articles, letters to the editor, opinions, commentaries, select communications, and corresponding articles.

The exclusion criteria were (1) articles not written in English, (2) articles that describe mothers with COVID-19 was diagnosed using methods other than RT-PCR, (3) articles with negative maternal RT-PCR results, (4) articles that do not report maternal age, (5) incompatible article type, including review articles, guidelines, and select communications, and (6) articles with an irrelevant topic.

### Review of Articles

Authors MC and ME extracted the following data from all included articles (Supplementary Materail), including (1) maternal COVID-19 status as indicated by RT-PCR tests, (2) neonatal COVID-19 status as indicated by RT-PCR test, and (3) corresponding neonatal Apgar scores.

### Data Analysis

Data were aggregated, and the median neonatal Apgar scores for 1 and 5 mins were calculated. Individual neonates were excluded from calculations if they were not tested, had inconclusive RT-PCR results, or if neither the RT-PCR result nor the Apgar score were reported for a given neonate. Only clear numerical values for the Apgar score were included, and the mean was taken for a range of numerical values.

### Statistical Analysis

Statistical analysis was performed using Mini Tab version 21.1.0. The data were presented as median, minimum values, maximum values, and sample sizes. For the purpose of statistical analysis, a χ^2^ test at a 95% confidence interval (α = 0.05) was applied. All reported two-tailed *p*-values were considered statistically significant when *p* < 0.05.

## Results

**Figure 1:**
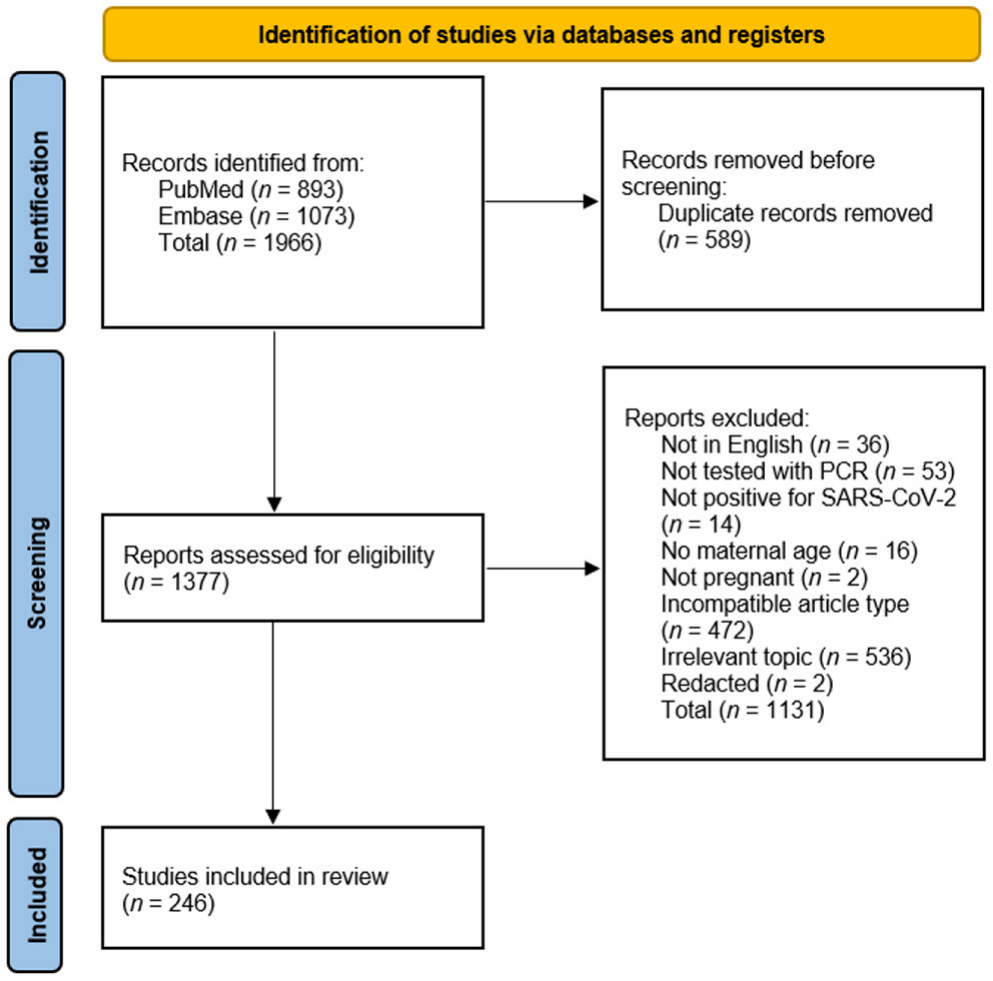
Study selection flowchart.

After the first stage of the review was completed, a total of 1,996 papers were identified. After removing duplicates, 1,377 papers were screened for eligibility according to the inclusion and exclusion criteria, and 1,131 papers were excluded. A total of 246 papers met the inclusion criteria and were included in the analyses (Figure 1).

The tallies revealed 663 total test-positive and test-negative neonates whose mothers had COVID-19. Neonates with positive RT-PCR results had median Apgar scores of 9 and 9 at 1 and 5 mins, respectively. Neonates with negative RT-PCR results had median Apgar scores of 8 and 9 at 1 and 5 mins, respectively (Table 1).

**Table 1:** Median apgar scores according to RT-PCR test.

**Maternal RT-PCR result**	**Neonatal RT-PCR results**	**Median apgar score at** **1 min (min, max, sample size)**	**Median apgar score at** **5 min (min, max, sample size)**
Positive	Positive	9 (0, 9.5, *n* = 236)	9 (0, 10, *n* = 222)
Positive	Negative	8 (0, 10, *n* = 423)	9 (0, 10, *n* = 426)

There were 22 (3%) and 11 (2%) neonates with a positive RT-PCR test who had Apgar scores under 7 at 1 and 5 mins, respectively. There were 29 (4%) and 11 (2%) neonates with negative RT-PCR tests who had Apgar scores below 7 at 1 and 5 mins, respectively (Table 2).

**Table 2:** Number of apgar scores under 7 at 1 and 5 mins according to neonatal RT-PCR result.

**Maternal RT-PCR result**	**Neonatal RT-PCR results**	**Apgar scores at** **1 min < 7 (%)**	**Apgar scores at 5 mins < 7 (%)**
Positive	Positive	22 (3)	11 (2)
Positive	Negative	29 (4)	11 (2)
*Chi*-squared test	Positive vs. Negative	*p* = 0.327, *χ^2^* = 0.961	*p* = 1, *χ^2^* = 0

## Discussion

### Apgar Scores During the COVID-19 Pandemic

Since the COVID-19 pandemic has adversely impacted maternal and neonatal outcomes, the pandemic has introduced a high degree of uncertainty for pregnant women and the outcomes of their pregnancies (2). A recent study indicated that the risk of having an Apgar score under 7 in a group of neonates whose mothers were diagnosed with COVID-19 was 25.4 times higher than the risk for a group of neonates whose mothers were not diagnosed with COVID-19, suggesting that maternal COVID-19 status impacts neonatal Apgar score (17).

However, given that comparable test-negative and test-positive neonates in this review had Apgar scores below 7, it is unlikely that neonatal COVID-19 status was responsible for low Apgar scores. Despite many asymptomatic pregnancies reported in the literature, many articles have suggested that severe maternal symptoms (including asphyxiation) prompting Cesarean section delivery may be a cause of low Apgar score in neonates (18–22). Specifically, one study implicated maternal SARS-CoV-2 infection as the major cause for placental damage and low Apgar score in neonates (21). Another common explanation for low Apgar score is preterm birth due to maternal COVID-19 complications, which results in developmentally premature neonates with lower well-being (23). Taken together, low Apgar scores are likely to be associated with severe maternal COVID-19 symptoms during delivery, rather an indicator of neonatal COVID-19 status (19–22).

In addition, a recent study indicated that the 1 and 5 min Apgar scores for neonates with either mild COVID-19 symptoms or severe or critical COVID-19 symptoms were comparable, which supports our findings that Apgar scores are poor indicators of the severity of COVID-19 in neonates (24). Similarly, another article found that there was no difference in the proportion of low Apgar scores between mothers with and without COVID-19 when universal testing schemes were implemented, while selective testing resulted in higher risk of low Apgar scores (25).

Agreeing with our findings, other studies have also shown that the numbers of neonates with 1 and 5 min Apgar scores under 7 were comparable regardless of whether the mothers had COVID-19 (26, 27). Moreover, other recent studies found that the percentage of neonates with Apgar scores under 7 was comparable before and during the COVID-19 pandemic (7, 8).

A comparison of neonates born pre- and post-implementation of COVID-19 labor and delivery guidelines showed no statistical differences in neonatal Apgar scores (28). Likewise, a recent review article revealed that the neonatal Apgar scores for infected neonates were similar to those for uninfected neonates (2). In addition, many neonates with positive RT-PCR results were clinically asymptomatic, showing no signs of complications due to COVID-19, and their lack of symptomology was reflected in their “reassuring” Apgar scores (29–31). Taken together, the data suggests that Apgar scores are poor indicators of COVID-19 status in neonates.

### Time Intervals of Apgar Scores

Although the Apgar test was designed to be administered by a medical professional at 1 and 5 mins of life after delivery and extended at 5 min intervals for neonates with Apgar scores below 7 to a maximum of 20 mins, there is disagreement in the literature regarding reporting times (4, 5). In particular, there has been criticism of the continued use of Apgar tests at 1 min. Initially, 1 min scores were developed as a clinical guideline for resuscitation, but recent protocols under the Neonatal Resuscitation Program have required resuscitation before 1 min, rendering a 1 min Apgar test obsolete (4). Moreover, Apgar scores at the first few minutes of life hold little long-term significance, as Apgar scores can fluctuate rapidly: even at 5 mins, neonates with “below normal” Apgar scores typically improve to reassuring Apgar scores by 10 mins (32).

However, other studies have pointed to clinical significance for 5 and 10 mins Apgar scores where some articles showed correlations between low Apgar scores at 5 mins with increase proportions of mortality, risk of cerebral palsy, and epilepsy (6). At the same time, low scores at 10 mins are associated with a higher risk of adverse outcomes (32). This disagreement about reporting times is evident in the literature; many recent papers only report 5 mins Apgar scores, omitting 1 min scores without providing explanation for their absence (7, 26, 28, 33). In keeping with the intended testing scheme, however, the vast majority of studies commonly report both 1 and 5 mins scores (2, 24, 27, 34).

### Apgar Score Cut-Off Value

Despite three distinct classifications of “reassuring,” “below normal,” and “critically low,” researchers have found limited clinical significance between certain scores, particularly between 6 as “below normal” and 7 as “reassuring.” (4, 35). The reported cut-off values for Apgar scores vary significantly among articles, deviating from the canonical 7 as “reassuring” to the use of atypical values, such as 4, 5, 8, and 9, which may reflect a desire to simplify values in reported research (24, 27, 36).

### Limitations

Although this scoping review synthesizes many articles in the existing literature, this work is subject to limitations. This scoping review excluded mothers with negative or suspected SARS-CoV-2 infection, but this exclusion may have resulted in missing articles with different outcomes. Similarly, by excluding neonatal Apgar scores in the absence of corresponding positive or negative RT-PCR tests, the sample size and the data are limited. Thus, the small sample size and the ongoing nature of the pandemic indicate that the results of the study should be interpreted with caution.

## Conclusion

The COVID-19 pandemic has introduced unprecedented challenges to health systems, and clinicians have responded by repurposing old tools to fill new needs. The Apgar test is one such tool that clinicians have used to provide some indication of neonatal COVID-19 status. The low proportion of Apgar scores below 7 indicates that low Apgar scores are likely to be associated with severe maternal COVID-19 symptoms during delivery rather than neonatal COVID-19. Therefore, this study indicated that Apgar scores are poor indicators of neonatal COVID-19 status. Based on these findings, we recommend that clinicians use alternative tests to indicate neonatal COVID-19 status. At the same time, the need remains to investigate the neonatal immune response to SARS-CoV-2 and elucidate the molecular mechanism responsible for neonatal well-being.

## Author Contributions

MC, CM, and ME conceived the study. All authors approved final manuscript.

## Conflict of Interest

The authors declare that the research was conducted in the absence of any commercial or financial relationships that could be construed as a potential conflict of interest.

## Publisher's Note

All claims expressed in this article are solely those of the authors and do not necessarily represent those of their affiliated organizations, or those of the publisher, the editors and the reviewers. Any product that may be evaluated in this article, or claim that may be made by its manufacturer, is not guaranteed or endorsed by the publisher.
