# Supplementary material for: Validity of Apgar Score as an Indicator of Neonatal SARS-CoV-2 Infection: A Scoping Review
Source: Front Med (Lausanne). 2022 Jan 11;8:782376. doi: 10.3389/fmed.2021.782376 (PMC8787091; doi:10.3389/fmed.2021.782376)
Supplement: Supplementary file 1 [file Data_Sheet_1.PDF]

Supplementary Information

1. Massa, H. *et al.* Belgian twins born with the Gamma variant of SARS-CoV-2: Transplacental versus intrapartum transmission? *European Journal of Obstetrics and Gynaceology and Reproductive Biology* **264**, 380–391 (2021).
2. Popescu, D. E. *et al.* A case of COVID-19 Pregnancy Complicated with Hydrops Fetalis and Intrauterine Death. *Medicina* **57**, 667–675 (2021).
3. Morales, H. S. G. *et al.* Vertical transmission: evidence of COVID-19 in a twin pregnancy. *JBRA Assisted Reproduction* (2021) doi:10.5935/1518-0557.20210058.
4. Edlow, A. G. *et al.* Assessment of Maternal and Neonatal SARS-CoV-2 Viral Load, Transplacental Antibody Transfer, and Placental Pathology in Pregnancies During the COVID-19 Pandemic. *JAMA Network Open* **3**, e2030455–e2030455 (2020).
5. Ahlberg, M. *et al.* Association of SARS-CoV-2 Test Status and Pregnancy Outcomes. *Journal of the American Medical Association* **324**, 1782–1785 (2020).
6. Rebutini, P. Z. *et al.* Association Between COVID-19 Pregnant Women Symptoms Severity and Placental Morphologic Features. *Frontiers in Immunology* **12**, 1893–1907 (2021).
7. Angelidou, A. *et al.* Association of Maternal Perinatal SARS-CoV-2 Infection with Neonatal Outcomes during the COVID-19 Pandemic in Massachusetts. *JAMA Network Open* **4**, e217523–e2175523 (2021).
8. Arora, D. *et al.* Assessment of materno-foetal transmission of SARS-CoV-2: A prospective pilot study. *Medical Journal Armed Forces India* **77**, S398–S403 (2021).
9. Liao, J. *et al.* Analysis of vaginal delivery outcomes among pregnant women in Wuhan, China during the COVID-19 pandemic. *International Journal of Gynecology and Obstetrics* **150**, 53–57 (2020).
10. Birindwa, E. K. *et al.* A case study of the first pregnant woman with COVID-19 in Bukavu, eastern Democratic Republic of the Congo. *Maternal Health, Neonatology and Perinatology* **7**, 1–6 (2021).
11. Alamar, I. *et al.* A Possible Case of Vertical Transmission of SARS-CoV-2 in a Newborn with Positive Placental In Situ Hybridization of SARS-CoV-2 RNA. *Journal of the Pediatric Infectious Disease* 636–639 (2020).
12. Huseynova, R. A. *et al.* A neonate born to an infected COVID-19 mother was tested positive just 24 hours after its birth. *Clinical Case Reports* **9**, 1954–1957 (2021).
13. Wang, S. *et al.* A case report of neonatal COVID-19 infection in China. *Clinical infectious diseases: an official publication of the Infectious Diseases Society of America* 1–14 (2020).
14. Peng, L. *et al.* Vertical transmission potential of SARS-CoV-2 from infected mother to twin neonates. *Future Virology* **16**, 379–382 (2021).
15. Sinaci, S. *et al.* Vertical transmission of SARS-CoV-2: A prospective cross-sectional study from a tertiary center. *Journal of Medical Virology* **93**, 5864–5872 (2021).
16. di Iorio, R., Bianchi, P., Bastianelli, C., Brosens, I. & Benagiano, G. Vertical transmission of SARS-CoV-2 infection in early pregnancy: what is the evidence? *Journal of Maternal-Fetal and Neonatal Medicine* 1–2 (2020) doi:10.1080/14767058.2020.1825671.
17. Rebello, C. M. *et al.* Vertical transmission of SARS-CoV-2 from infected pregnant mother to the neonate detected by cord blood real-time polymerase chain reaction (RT-PCR). *Pediatric Research* **89**, 1592–1593 (2021).
18. Sukhikh, G. *et al.* Vertical transmission of SARS-CoV-2 in second trimester associated with severe neonatal pathology. *Viruses* **13**, 447–458 (2021).
19. Singh, M. V. *et al.* Vertical Transmission of SARS-CoV-2 from an Asymptomatic Pregnant Woman in India. *Journal of Tropical Pediatrics* **67**, fmaa048-fma050 (2021).
20. Maeda, M. de F. Y. *et al.* Vertical transmission of SARS-CoV2 during pregnancy: A high-risk cohort. *Prenatal Diagnosis* **41**, 998–1008 (2021).

21. Pulinx, B. *et al.* Vertical transmission of SARS-CoV-2 infection and preterm birth. *European Journal of Clinical Microbiology and Infectious Diseases* **39**, 2441–2445 (2020).
22. Lopez Salinas, D. *et al.* Vertical Transmission of COVID-19 in Children of Sero-positive Mothers to SARS-CoV-2 in Southeast Mexico: A Case Report. *Respiratory Case Reports* **10**, 1–7 (2021).
23. Moreno, S. C., To, J., Chun, H. & Ngai, I. M. Vertical Transmission of COVID-19 to the Neonate. *Infectious Diseases in Obstetrics and Gynecology* **2020**, (2020).
24. He, Z. *et al.* Vertical transmission and kidney damage in newborns whose mothers had coronavirus disease 2019 during pregnancy. *International Journal of Antimicrobial Agents* **57**, (2021).
25. Masmejan, S. *et al.* Vertical transmission and materno-fetal outcomes in 13 patients with coronavirus disease 2019. *Clinical Microbiology and Infection* **26**, 1585–1587 (2020).
26. Thapa, B., Acharya, S. & Karki, S. Vertical Transmission of COVID-19: A Case Report and Review of Literature. *Journal of Nepal Health Research Council* **19**, 203–205 (2021).
27. Cao, D., Chen, M., Peng, M., Yin, H. & Sun, G. Vaginal delivery in women with COVID-19: Report of two cases. *BMC Pregnancy and Childbirth* **20**, (2020).
28. Xiong, X. *et al.* Vaginal delivery report of a healthy neonate born to a convalescent mother with COVID-19. *Journal of Medical Virology* **92**, 1657–1659 (2020).
29. Rottenstreich, A. *et al.* Vaginal delivery in SARS-CoV-2-infected pregnant women in Israel: a multicenter prospective analysis. *Archives of Gynecology and Obstetrics* **303**, 1401–1405 (2021).
30. Ferrazzi, E. *et al.* Vaginal delivery in SARS-CoV-2-infected pregnant women in Northern Italy: a retrospective analysis. *An International Journal of Obstetrics and Gynaceology* **127**, 1–6 (2020).
31. Polónia-Valente, R. *et al.* Vaginal delivery in a woman infected with SARS-CoV-2 – The first case reported in Portugal. *European Journal of Obstetrics and Gynecology and Reproductive Biology* **250**, 253–254 (2020).
32. Fenizia, C. *et al.* Unlikely SARS-CoV-2 Transmission During Vaginal Delivery. *Reproductive Sciences* **28**, 2939–2941 (2021).
33. Sagheb, S., Lamsehchi, A., Jafary, M., Atef-Yekta, R. & Sadeghi, K. Two seriously ill neonates born to mothers with COVID-19 pneumonia- a case report. *Italian Journal of Pediatrics* **46**, (2020).
34. Schwartz, D. A. *et al.* Spectrum of neonatal COVID-19 in Iran: 19 infants with SARS-CoV-2 perinatal infections with varying test results, clinical findings and outcomes. *Journal of Maternal-Fetal and Neonatal Medicine* 1–10 (2020) doi:10.1080/14767058.2020.1797672.
35. Ganor Paz, Y. *et al.* The association between SARS-CoV-2 infection and late pregnancy loss. *International Journal of Gynecology & Obstetrics* 1–2 (2021) doi:10.1002/ijgo.14025.
36. Ramírez-Rosas, A. *et al.* Study of perinatal transmission of SARS-CoV-2 in a Mexican public hospital. *International Journal of Infectious Diseases* **113**, 225–232 (2021).
37. Rubio Lorente, A. M. *et al.* Study of amniotic fluid in pregnant women infected with SARS-CoV-2 in first and second trimester. Is there evidence of vertical transmission? *Journal of Maternal-Fetal and Neonatal Medicine* 1–3 (2020) doi:10.1080/14767058.2020.1811669.
38. Sanchez, J. *et al.* Severe acute respiratory syndrome coronavirus 2 detected in placentas of 2 coronavirus disease 2019–positive asymptomatic pregnant women—case report. *AJOG Global Reports* **1**, 100001 (2021).
39. Carbayo-Jiménez, T. *et al.* Severe Acute Respiratory Syndrome Coronavirus 2 Vertical Transmission from an Asymptomatic Mother. *Pediatric Infectious Disease Journal* **40**, E115–E117 (2021).
40. Yasa, B. *et al.* Severity of Maternal SARS-CoV-2 Infection in Pregnancy Predicts Neonatal Outcomes. *American Journal of Perinatology* (2021) doi:10.1055/s-0041-1733783.
41. Schoenmakers, S. *et al.* Severe acute respiratory syndrome coronavirus 2 placental infection and inflammation leading to fetal distress and neonatal multi-organ failure in an asymptomatic woman. *Journal of the Pediatric Infectious Diseases Society* **10**, 556–561 (2021).

42. Hu, X. *et al.* Severe Acute Respiratory Syndrome Coronavirus 2 (SARS-CoV-2) Vertical Transmission in Neonates Born to Mothers With Coronavirus Disease 2019 (COVID-19) Pneumonia. *Obstetrics & Gynecology* **136**, 1–3 (2020).
43. Villalaín, C. *et al.* Seroprevalence analysis of SARS-CoV-2 in pregnant women along the first pandemic outbreak and perinatal outcome. *PLoS ONE* **15**, e0243029-30243041 (2020).
44. Hopwood, A. J. *et al.* Severe Acute Respiratory Syndrome Coronavirus-2 Pneumonia in a Newborn Treated with Remdesivir and Coronavirus Disease 2019 Convalescent Plasma. *Journal of the Pediatric Infectious Diseases Society* **10**, 691–694 (2021).
45. Gupta, P., Kumar, S. & Sharma, S. S. SARS-CoV-2 prevalence and maternal-perinatal outcomes among pregnant women admitted for delivery: Experience from COVID-19-dedicated maternity hospital in Jammu, Jammu and Kashmir (India). *Journal of Medical Virology* **93**, 5505–5514 (2021).
46. Moresi, S. *et al.* SARS-CoV-2 Infection in Pregnancy: Clinical Signs, Placental Pathology, and Neonatal Outcome—Implications for Clinical Care. *Frontiers in Medicine* **8**, (2021).
47. Celik, E. *et al.* Placental deficiency during maternal SARS-CoV-2 infection. *Placenta* **117**, 47–56 (2022).
48. Dhawan, S. & Pandey, M. SARS-CoV-2 Vertical Transmission: Rare But a Potential Possibility. *Indian Journal of Pediatrics* **88**, 277 (2021).
49. de Vasconcelos Gaspar, A. & Santos Silva, I. SARS-CoV-2 in Pregnancy-The First Wave. *Medicina* **57**, (2021).
50. Steffen, H. A. *et al.* SARS-CoV-2 Infection during Pregnancy in a Rural Midwest All-delivery Cohort and Associated Maternal and Neonatal Outcomes. *American Journal of Perinatology* **38**, 614–621 (2021).
51. Mourad, M. *et al.* Placental response to maternal SARS-CoV-2 infection. *Scientific Reports* **11**, (2021).
52. Barbero, P. *et al.* SARS-CoV-2 in pregnancy: characteristics and outcomes of hospitalized and non-hospitalized women due to COVID-19. *Journal of Maternal-Fetal and Neonatal Medicine* 1–7 (2020) doi:10.1080/14767058.2020.1793320.
53. Morotti, D. *et al.* Molecular pathology analysis of sars-cov-2 in syncytiotrophoblast and hofbauer cells in placenta from a pregnant woman and fetus with covid-19. *Pathogens* **10**, (2021).
54. Lopian, M. *et al.* Safety of vaginal delivery in women infected with COVID-19. *Pediatrics and Neonatology* **62**, 90–96 (2021).
55. di Mascio, D. *et al.* Risk factors associated with adverse fetal outcomes in pregnancies affected by Coronavirus disease 2019 (COVID-19): A secondary analysis of the WAPM study on COVID-19. *Journal of Perinatal Medicine* **48**, 950–958 (2020).
56. Zhang, P. *et al.* Maternal, neonatal and placental characteristics of SARS-CoV-2 positive mothers. *Journal of Maternal-Fetal and Neonatal Medicine* 1–9 (2021) doi:10.1080/14767058.2021.1892637.
57. Vivanti, A. J. *et al.* Retrospective description of pregnant women infected with severe acute respiratory syndrome coronavirus 2, France. *Emerging Infectious Diseases* **26**, 2069–2076 (2020).
58. Perrone, S. *et al.* Report of a series of healthy term newborns from convalescent mothers with covid-19. *Acta Biomedica* **91**, 251–255 (2020).
59. Alipour, Z. *et al.* Relationship between coronavirus disease 2019 in pregnancy and maternal and fetal outcomes: Retrospective analytical cohort study. *Midwifery* **102**, (2021).
60. Daniel, K., Goli, K. & Sargent, A. Repeat cesarean section in a COVID-19 positive mother in the United States. *SAGE Open Medical Case Reports* **8**, 2050313X2094553 (2020).
61. Karade, S., Vishal, A. K., Sen, S., Bewal, N. & Gupta, R. M. Probable vertical transmission of severe acute respiratory syndrome coronavirus 2 infection from mother to neonate. *Medical Journal Armed Forces India* **77**, S490–S493 (2021).
62. Kirtsman MDCM, M. *et al.* Probable congenital SARS-CoV-2 infection in a neonate born to a woman with active SARS-CoV-2 infection. *CMAJ* **15**, 647–651 (2020).

63. Cardona-Pérez, J. A. *et al.* Prevalence, clinical features, and outcomes of SARS-CoV-2 infection in pregnant women with or without mild/moderate symptoms: Results from universal screening in a tertiary care center in Mexico City, Mexico. *PLoS ONE* **16**, (2021).
64. Pessoa, F. S. *et al.* Probable vertical transmission identified within six hours of life. *Revista da Associacao Medica Brasileira* **66**, 1621–1624 (2020).
65. Mendoza-Hernández, M. *et al.* Probable Case of Vertical Transmission of SARS-CoV-2 in a Newborn in Mexico. *Neonatology* **118**, 364–367 (2021).
66. Sebastião, C. S. *et al.* Prevalence and Risk Factors of SARS-CoV-2 Infection among Parturients and Newborns from Luanda, Angola. *Pathogens* **10**, 1494 (2021).
67. Cosma, S. *et al.* Preterm birth is not associated with asymptomatic/mild SARS-CoV-2 infection per se: Pre-pregnancy state is what matters. *PLoS ONE* **16**, (2021).
68. Zamaniyan, M. *et al.* Preterm delivery, maternal death, and vertical transmission in a pregnant woman with COVID-19 infection. *Prenatal Diagnosis* **40**, 1759–1761 (2020).
69. Gandhi, A. M., Ganatra, A. M. & Tank, P. Preliminary Results from the FOGSI's National Registry on Pregnancy with COVID-19. *Journal of Obstetrics and Gynecology of India* **71**, 361–368 (2021).
70. Kumari, K. *et al.* Pregnancy outcomes and vertical transmission capability of SARS-CoV-2 infection among asymptomatic females: A cross-sectional study in a tertiary care rural hospital. *Journal of Family Medicine and Primary Care* **10**, 3247 (2021).
71. Sileo, F. G. *et al.* Pregnant woman infected by Coronavirus disease (COVID-19) and calcifications of the fetal bowel and gallbladder. *Minerva Obstetrics and Gynecology* **73**, 121–124 (2021).
72. Marca, L. A. Pregnant woman infected by Coronavirus disease (COVID-19) and calcifications of the fetal bowel and gallbladder. *Obstetrics and Gynecology* **73**, 121–124 (2020).
73. Prabhu, M. *et al.* Pregnancy and postpartum outcomes in a universally tested population for SARS-CoV-2 in New York City: a prospective cohort study. *BJOG: An International Journal of Obstetrics and Gynaecology* **127**, 1548–1556 (2020).
74. Schwartz, D. A. & Graham, A. L. Potential maternal and infant outcomes from coronavirus 2019-NCOV (SARS-CoV-2) infecting pregnant women: Lessons from SARS, MERS, and other human coronavirus infections. *Viruses* **12**, (2020).
75. Marzollo, R. *et al.* Possible Coronavirus Disease 2019 Pandemic and Pregnancy: Vertical Transmission Is Not Excluded. *Pediatric Infectious Disease Journal* E261–E262 (2020) doi:10.1097/INF.0000000000002816.
76. Parsa, Y. *et al.* Possible Vertical Transmission of COVID-19 to the Newborn; a Case Report. *Archives of Academic Emergency Medicine* **9**, 1–3 (2020).
77. Naseh, A. & Ashrafzadeh, S. Possible Vertical Transmission From an Unsuspected SARS-CoV-2-Infected Mother to Her Newborn. *Cureus* **13**, (2021).
78. Bandyopadhyay, T., Sharma, A., Kumari, P., Maria, A. & Choudhary, R. Possible Early Vertical Transmission of COVID-19 from an Infected Pregnant Female to Her Neonate: A Case Report. *Journal of Tropical Pediatrics* **67**, (2021).
79. di Guardo, F. *et al.* Poor maternal–neonatal outcomes in pregnant patients with confirmed SARS-Cov-2 infection: analysis of 145 cases. *Archives of Gynecology and Obstetrics* **303**, 1483–1488 (2021).
80. Sweeney, I., al Assaf, N. & Khan, R. Placental Swab in Supporting Diagnosis of Vertical Transmission in SARS-CoV-2 Positive Mothers. *Ir Med J* **114**, 409–418 (2020).
81. Hsu, A. L. *et al.* Placental SARS-CoV-2 in a pregnant woman with mild COVID-19 disease. *Journal of Medical Virology* **93**, 1038–1044 (2021).
82. Shende, P. *et al.* Persistence of SARS-CoV-2 in the first trimester placenta leading to transplacental transmission and fetal demise from an asymptomatic mother Running Title: Placental infection of SARS-CoV-2 and fetal demise. *Human Reproduction* **36**, 889–906 (2021).
83. Ioscovich, A. *et al.* Peripartum anesthetic management of women with SARS-CoV-2 infection in eight medical centers across three European countries: prospective cohort observation study.

Journal of Maternal-Fetal and Neonatal Medicine 1–8 (2021) doi:10.1080/14767058.2021.1937105.
84. Fan, C. *et al.* Perinatal Transmission of COVID-19 Associated SARS-CoV-2: Should We Worry? *Clinical Infectious Diseases* **72**, 862–864 (2020).
85. Mehta, H. *et al.* Novel coronavirus-related acute respiratory distress syndrome in a patient with twin pregnancy: A case report. *Case Reports in Women's Health* **27**, (2020).
86. Tallarek, A. C. *et al.* Inefficient Placental Virus Replication and Absence of Neonatal Cell-Specific Immunity Upon Sars-CoV-2 Infection During Pregnancy. *Frontiers in Immunology* **12**, (2021).
87. Salvatore, C. M. *et al.* Neonatal management and outcomes during the COVID-19 pandemic: an observation cohort study. *The Lancet Child and Adolescent Health* **4**, 721–727 (2020).
88. Wu, Y. T. *et al.* Neonatal outcome in 29 pregnant women with COVID-19: A retrospective study in Wuhan, China. *PLoS Medicine* **17**, (2020).
89. Urban, A. & Dyrda, M. Mother and neonate suffering from COVID-19 infection. Is there any risk of vertical transmission? A case report. *Ginekologia Polska* **92**, 701–703 (2021).
90. Resta, L. *et al.* Is the first of the two born saved? A rare and dramatic case of double placental damage from SARS-CoV-2. *Viruses* **13**, (2021).
91. Saccone, G. *et al.* Maternal and perinatal outcomes of pregnant women with SARS-CoV-2 infection. *Ultrasound in Obstetrics and Gynecology* **57**, 232–241 (2021).
92. Blasco Santana, L. *et al.* Maternal and perinatal outcomes and placental pathologic examination of 29 SARS-CoV-2 infected patients in the third trimester of gestation. *Journal of Obstetrics and Gynaecology Research* **47**, 2131–2139 (2021).
93. Prochaska, E., Jang, M. & Burd, I. COVID-19 in pregnancy: Placental and neonatal involvement. *American Journal of Reproductive Immunology* **84**, (2020).
94. Remaeus, K. *et al.* Characteristics and short-term obstetric outcomes in a case series of 67 women test-positive for SARS-CoV-2 in Stockholm, Sweden. *Acta Obstetricia et Gynecologica Scandinavica* **99**, 1626–1631 (2020).
95. Valdespino-Vázquez, M. Y. *et al.* Fetal and placental infection with SARS-CoV-2 in early pregnancy. *Journal of Medical Virology* **93**, 4480–4487 (2021).
96. Colson, A. *et al.* Clinical and in Vitro Evidence against Placenta Infection at Term by Severe Acute Respiratory Syndrome Coronavirus 2. *American Journal of Pathology* **191**, 1610–1623 (2021).
97. Lesnic, A. *et al.* Can SARS-CoV-2 induce uterine vascular anomalies and poor contractile response?—A case report. *Medicina* **57**, 670–678 (2021).
98. Shaiba, L. A. *et al.* Case Report: Neonatal Multi-System Inflammatory Syndrome Associated With SARS-CoV-2 Exposure in Two Cases From Saudi Arabia. *Frontiers in Pediatrics* **9**, (2021).
99. Alwardi, T. H. *et al.* Is Vertical Transmission of SARS-CoV-2 Infection Possible in Preterm Triplet Pregnancy? A Case Series. *Pediatric Infectious Disease Journal* E456–E458 (2020) doi:10.1097/INF.0000000000002926.
100. Alzamora, M. C. *et al.* Severe COVID-19 during Pregnancy and Possible Vertical Transmission. *American Journal of Perinatology* **37**, 861–865 (2020).
101. Filimonovic, D. *et al.* Intrauterine transfusion in COVID-19 positive mother vertical transmission risk assessment. *European Journal of Obstetrics and Gynecology and Reproductive Biology* **252**, 617–618 (2020).
102. Ogamba, I. *et al.* Initial review of pregnancy and neonatal outcomes of pregnant women with COVID-19 infection. *Journal of Perinatal Medicine* **49**, 263–268 (2021).
103. de Nardo, M. C. *et al.* Impact of joint management of a COVID-19 mother and her newborn on the virus transmission: a case report. *Virology Journal* **18**, 1–4 (2021).
104. Chen, Y. *et al.* Infants Born to Mothers With a New Coronavirus (COVID-19). *Frontiers in Pediatrics* **8**, (2020).

105. Conti, M. G. *et al.* Immune Response of Neonates Born to Mothers Infected With SARS-CoV-2. *JAMA network open* **4**, e2132563 (2021).
106. Easterlin, M. C., de Beritto, T., Yeh, A. M., Wertheimer, F. B. & Ramanathan, R. Extremely Preterm Infant Born to a Mother With Severe COVID-19 Pneumonia. *Journal of Investigative Medicine High Impact Case Reports* **8**, (2020).
107. Rosen, H. *et al.* Fetal and perinatal outcome following first and second trimester covid-19 infection: Evidence from a prospective cohort study. *Journal of Clinical Medicine* **10**, (2021).
108. Kulkarni, R. *et al.* Early-onset symptomatic neonatal COVID-19 infection with high probability of vertical transmission. *Infection* **49**, 339–343 (2021).
109. Agarwal, M., Basumatary, S., Bhusan, D. & Pati, B. K. Detection of severe acute respiratory syndrome corona virus 2 in cervico-vaginal secretion of COVID-19-affected female: A prospective observational study from India. *SAGE Open Medicine* **9**, 205031212110229 (2021).
110. Hazari, K. S. *et al.* Covid-19 infection in pregnant women in Dubai: a case-control study. *BMC Pregnancy and Childbirth* **21**, (2021).
111. Rashidian, T. *et al.* Death of a neonate with suspected coronavirus disease 2019 born to a mother with coronavirus disease 2019 in Iran: A case report. *Journal of Medical Case Reports* **14**, (2020).
112. Zaigham, M. & Andersson, O. Maternal and perinatal outcomes with COVID-19: A systematic review of 108 pregnancies. *Acta Obstetricia et Gynecologica Scandinavica* **99**, 823–829 (2020).
113. Palalioglu, R. M., Mahammadaliyeva, A., Erbiyik, H. I. & Muhcu, M. COVID-19 in third trimester may not be as scary as you think, it can be innocent: Evaluating vertical transmission from a COVID-19 positive asymptomatic pregnant woman with early membrane rupture. *Journal of Obstetrics and Gynaecology Research* **47**, 838–842 (2021).
114. Engjom, H. *et al.* COVID-19 in pregnancy—characteristics and outcomes of pregnant women admitted to hospital because of SARS-CoV-2 infection in the Nordic countries. *Acta Obstetricia et Gynecologica Scandinavica* **100**, 1611–1619 (2021).
115. Volpe, N. *et al.* COVID-19 in pregnancy: where are we now? *Journal of Perinatal Medicine* **49**, 637–642 (2021).
116. Askary, E. *et al.* Coronavirus disease 2019 (COVID-19) manifestations during pregnancy in all three trimesters: A case series. *International Journal of Reproductive BioMedicine* **19**, 191–204 (2021).
117. Rivera-Hernandez, P. *et al.* Coronavirus Disease 2019 in a Premature Infant: Vertical Transmission and Antibody Response or Lack Thereof. *AJP Reports* **10**, E224–E227 (2020).
118. Correia, C. R. *et al.* Congenital SARS-CoV-2 Infection in a Neonate with Severe Acute Respiratory Syndrome. *Pediatric Infectious Disease Journal* E439–E443 (2020) doi:10.1097/INF.0000000000002941.
119. Mok, T. *et al.* Complicated Monochorionic-Diamniotic Twins in a Pregnant Woman with COVID-19 in the Second Trimester. *American Journal of Perinatology* **38**, 747–752 (2021).
120. Al-Matary, A. *et al.* Clinical outcomes of maternal and neonate with COVID-19 infection – Multicenter study in Saudi Arabia. *Journal of Infection and Public Health* **14**, 702–708 (2021).
121. Farhadi, R., Mehrpisheh, S., Ghaffari, V., Haghshenas, M. & Ebadi, A. Clinical course, radiological findings and late outcome in preterm infant with suspected vertical transmission born to a mother with severe COVID-19 pneumonia: a case report. *Journal of Medical Case Reports* **15**, 1–5 (2021).
122. Shmakov, R. G. *et al.* Clinical course of novel COVID-19 infection in pregnant women. *Journal of Maternal-Fetal and Neonatal Medicine* (2020) doi:10.1080/14767058.2020.1850683.
123. Arakaki, T. *et al.* Clinical characteristics of pregnant women with COVID-19 in Japan: a nationwide questionnaire survey. *BMC Pregnancy and Childbirth* **21**, (2021).
124. Yang, P. *et al.* Clinical characteristics and risk assessment of newborns born to mothers with COVID-19. *Journal of Clinical Virology* **127**, 104356–104362 (2020).
125. Liu, W. *et al.* Clinical characteristics of 19 neonates born to mothers with COVID-19. *Frontiers of Medicine* **14**, 193–198 (2020).

126. Debelenko, L. *et al.* Trophoblast damage with acute and chronic intervillositis: disruption of the placental barrier by severe acute respiratory syndrome coronavirus 2. *Human Pathology* **109**, 69–79 (2021).
127. Rehana, R., Jamoor, K. & Huda, N. SARS-CoV-2-Induced Emergency Cesarian Section. *CCM Journal* **49**, 123–123 (2020).
128. Gee, S. *et al.* The legacy of maternal SARS-CoV-2 infection on the immunology of the neonate. *Nature Immunology* 1–13 (2021) doi:10.1038/s41590-021-01049-2.
129. Smithgall, M. C. *et al.* Third-trimester placentas of severe acute respiratory syndrome coronavirus 2 (SARS-CoV-2)-positive women: histomorphology, including viral immunohistochemistry and in-situ hybridization. *Histopathology* **77**, 994–999 (2020).
130. Linehan, L. *et al.* SARS-CoV-2 placentitis: An uncommon complication of maternal COVID-19. *Placenta* **104**, 261–266 (2021).
131. Choobdar, F. A. *et al.* Transplacental transmission of SARS-CoV-2 infection: A case report from Iran. *Archives of Pediatric Infectious Diseases* **9**, (2021).
132. Cribiù, F. M. *et al.* Severe SARS-CoV-2 placenta infection can impact neonatal outcome in the absence of vertical transmission. *Journal of Clinical Investigation* **131**, (2021).
133. Wang, X. *et al.* A case of 2019 Novel Coronavirus in a pregnant woman with preterm delivery. 1–9 (2020).
134. Bae, J. G. *et al.* A case of delivery of a pregnant woman with COVID-19 infection in Daegu, Korea. *Obstetrics and Gynecology Science* **63**, 745–749 (2020).
135. Patane, L., Morotti, D. & Giunta, M. Vertical transmission of coronavirus disease 2019: severe acute respiratory syndrome coronavirus 2 RNA on the fetal side of the placenta in pregnancies with coronavirus disease 2019 positive mothers and neonates at birth. *AJOG MFM* **2**, 100145 (2020).
136. Peng, Z. *et al.* Unlikely SARS-CoV-2 vertical transmission from mother to child: A case report. *Journal of Infection and Public Health* **13**, 818–820 (2020).
137. Mejía Jiménez, I. *et al.* Umbilical cord clamping and skin-to-skin contact in deliveries from women positive for SARS-CoV-2: a prospective observational study. *BJOG: an international journal of obstetrics and gynaecology* **128**, 908–915 (2021).
138. Soto-Torres, E., Hernandez-Andrade, E., Huntley, E., Mendez-Figueroa, H. & Blackwell, S. C. Ultrasound and Doppler findings in pregnant women with SARS-CoV-2 infection. *Ultrasound in Obstetrics and Gynecology* **58**, 111–120 (2021).
139. Kapadia, S. N. *et al.* Study of pregnancy with covid-19 and its clinical outcomes in a tertiary care teaching hospital in Western India. *Journal of SAFOG* **13**, 125–130 (2021).
140. Mehaffey, J. H. *et al.* Successful vertical transmission of SARS-CoV-2 antibodies after maternal vaccination. *Birth* **48**, 451–452 (2021).
141. Douedi, S., Albayati, A., Alfraji, N., Mazahir, U. & Costanzo, E. Successful maternal and fetal outcomes in COVID-19 pregnant women: An institutional approach. *American Journal of Case Reports* **21**, 1–3 (2020).
142. Rodrigues, M., Gasparinho, G., Sepulvedda, F. & Matos, T. Signs suggestive of congenital SARS-CoV-2 infection with intrauterine fetal death: A case report. *European Journal of Obstetrics and Gynecology and Reproductive biology* **256**, 502–526 (2021).
143. Carrasco, I. *et al.* SARS-COV-2 infection in pregnant women and newborns in a Spanish cohort (GESNEO-COVID) during the first wave. *BMC Pregnancy and Childbirth* **21**, (2021).
144. Demirjian, A. *et al.* Probable Vertical Transmission of SARS-CoV-2 Infection. *Pediatric Infectious Disease Journal* **39**, 3257-e260 (2020).
145. Akdemir, Y. *et al.* Probable viremia and positive placental swabs for SARS-CoV-2 in a preterm pregnant woman with mild COVID-19. *Journal of Medical Virology* **93**, 6788–6793 (2021).
146. Mattar, C. N. *et al.* Pregnancy Outcomes in COVID-19: A Prospective Cohort Study in Singapore. *Annals of the Academy of Medicine, Singapore* **49**, 857–869 (2020).

147. Spir, P. R. N. *et al.* Probable Vertical Transmission of SARS-CoV-2 Infection in the Countryside of São Paulo State, Brazil: Case Report. *The Journal of Pediatric Research* **8**, 367–369 (2021).
148. Brien, M. E. *et al.* Pandemic stress and SARS-CoV-2 infection are associated with pathological changes at the maternal-fetal interface. *Placenta* **115**, 37–44 (2021).
149. Mao, Q., Chu, S., Shapiro, S., Bliss, J. M. & de Paepe, M. E. Increased placental expression of angiotensin-converting enzyme 2, the receptor of SARS-CoV-2, associated with hypoxia in twin anemia-polycythemia sequence (TAPS). *Placenta* **105**, 7–13 (2021).
150. Li, N. *et al.* Maternal and Neonatal Outcomes of Pregnant Women with Coronavirus Disease 2019 (COVID-19) Pneumonia: A Case-Control Study. *Clinical Infectious Diseases* **71**, 2035–2041 (2020).
151. Yang, R. *et al.* Pregnant women with COVID-19 and risk of adverse birth outcomes and maternal-fetal vertical transmission: a population-based cohort study in Wuhan, China. *BMC Medicine* **18**, 1–17 (2020).
152. Adhikari, E. H. *et al.* Pregnancy Outcomes among Women with and without Severe Acute Respiratory Syndrome Coronavirus 2 Infection. *JAMA Network Open* **3**, e2029256–e2029256 (2020).
153. Jang, W. K. *et al.* Pregnancy outcome, antibodies and placental pathology in sars-cov-2 infection during early pregnancy. *International Journal of Environmental Research and Public Health* **18**, (2021).
154. Joudi, N., Henkel, A., Lock, W. & Lyell, D. Preeclampsia treatment in severe acute respiratory syndrome coronavirus 2. *AJOG MFM* **2**, 1–3 (2020).
155. Sahin, D., Tanacan, A., Webster, S. & Moraloglu, O. Pregnancy and COVID-19: prevention, vaccination, therapy and beyond. *Turkish Journal of Medical Sciences* (2021) doi:10.3906/sag-2106-134.
156. Salehipour, H., Yazdi, M. E., Abzevar, M. T., Shomoossi, N. & Rad, M. Possibility of intrauterine vertical transmission of coronavirus disease 2019 (COVID-19): A case report from Iran. *International Journal of Preventive Medicine* **12**, (2021).
157. Biringer, K. *et al.* Placental pathology concerning sudden foetal demise in sars-cov-2 positive asymptomatic pregnant female. *Biomedical Papers* **165**, 328–331 (2021).
158. Arinkan, S. A., Dallı Alper, E. C., Topcu, G. & Muhcu, M. Perinatal outcomes of pregnant women having SARS-CoV-2 infection. *Taiwanese Journal of Obstetrics and Gynecology* **60**, 1043–1046 (2021).
159. Sharma, R. *et al.* Perinatal outcome and possible vertical transmission of coronavirus disease 2019: Experience from North India. *Clinical and Experimental Pediatrics* **64**, 239–246 (2021).
160. Dumitriu, D. *et al.* Outcomes of Neonates Born to Mothers with Severe Acute Respiratory Syndrome Coronavirus 2 Infection at a Large Medical Center in New York City. *JAMA Pediatrics* **175**, 157–167 (2021).
161. Cavaliere, A. F., Marchi, L., Aquilini, D., Brunelli, T. & Vasarri, P. L. Passive immunity in newborn from SARS-CoV-2-infected mother. *Journal of Medical Virology* **93**, 1810–1813 (2021).
162. Ghema, K. *et al.* Outcomes of newborns to mothers with COVID-19. *Infectious Diseases Now* **51**, 435–439 (2021).
163. Sutton, D., Fuchs, K., D'Alton, M. & Goffman, D. Universal Screening for SARS-CoV-2 in Women Admitted for Delivery. *New England Journal of Medicine* **382**, 2163–2164 (2020).
164. Niermeyer, S., Little, G. A., Singhal, N. & Keenan, W. J. A short history of helping babies breathe: Why and how, then and now. *Pediatrics* **146**, (2020).
165. Munian, D., Das, R., Hazra, A. & Ray, S. Outcome of Neonates Born to COVID-Positive Women at 6 Months of Age. *Indian Pediatrics* **58**, 853–856 (2021).
166. Cosma, S. *et al.* Obstetric and neonatal outcomes after SARS-CoV-2 infection in the first trimester of pregnancy: A prospective comparative study. *Journal of Obstetrics and Gynaecology Research* (2021) doi:10.1111/jog.15105.

167. Alhamoud, A. H., Matary, F., Bukhari, S., Kelantan, M. & Bajahzer, M. Outcomes of an In Vitro Fertilization Pregnancy With COVID-19 and the Perinatal Outcome in Riyadh, Saudi Arabia. *Cureus* (2020) doi:10.7759/cureus.12296.
168. Trahan, M. J. *et al.* Obstetrical and Newborn Outcomes Among Patients With SARS-CoV-2 During Pregnancy. *Journal of Obstetrics and Gynaecology Canada* **43**, 888-892.e1 (2021).
169. Lv, Y. *et al.* No intrauterine vertical transmission in pregnancy with COVID-19: A case report. *Journal of Infection and Chemotherapy* **26**, 1313–1315 (2020).
170. Patil, U. P. *et al.* Newborns of COVID-19 mothers: short-term outcomes of colocating and breastfeeding from the pandemic's epicenter. *Journal of Perinatology* **40**, 1455–1458 (2020).
171. Grimminck, K. *et al.* No evidence of vertical transmission of SARS-CoV-2 after induction of labour in an immune-suppressed SARS-CoV-2-positive patient. *BMJ Case Reports* **13**, (2020).
172. Chen, Y. C. *et al.* Neutralization antibody titers against SARS-CoV-2 in an infant born to a mother with COVID-19. *Pediatrics and Neonatology* **62**, 661–663 (2021).
173. Nayak, M. K. *et al.* Neonatal outcomes of pregnant women with COVID-19 in a developing country setup. *Pediatrics and Neonatology* **62**, 499–505 (2021).
174. Shlomai, N. *et al.* Neonatal SARS-CoV-2 Infections in Breastfeeding Mothers. *Pediatrics* **147**, (2021).
175. Marín Gabriel, M. A. *et al.* Multicentre Spanish study found no incidences of viral transmission in infants born to mothers with COVID-19. *Acta Paediatrica, International Journal of Paediatrics* **109**, 2302–2308 (2020).
176. Gabriel, M. Á. M., Martínez, A. M. M., Martínez, M. E. M. & Pedroche, J. A. Negative Transmission of SARS-CoV-2 to Hand-Expressed Colostrum from SARS-CoV-2-Positive Mothers. *Breastfeeding Medicine* **15**, 492–494 (2020).
177. Sabharwal, V. *et al.* Mother-Infant Dyads with COVID-19 at an Urban, Safety-Net Hospital: Clinical Manifestations and Birth Outcomes. *American Journal of Perinatology* **38**, 741–746 (2021).
178. Budhram, S. *et al.* Maternal characteristics and pregnancy outcomes of hospitalized pregnant women with SARS-CoV-2 infection in South Africa: An International Network of Obstetric Survey Systems-based cohort study. *International Journal of Gynecology and Obstetrics* **155**, 455–465 (2021).
179. Marín Gabriel, M. A. *et al.* Maternal, Perinatal and Neonatal Outcomes with COVID-19: A Multicenter Study of 242 Pregnancies and Their 248 Infant Newborns during Their First Month of Life. *Pediatric Infectious Disease Journal* E393–E397 (2020) doi:10.1097/INF.0000000000002902.
180. Douglass, K. M. *et al.* Maternal-Neonatal Dyad Outcomes of Maternal COVID-19 Requiring Extracorporeal Membrane Support: A Case Series. *American Journal of Perinatology* **38**, 082–087 (2021).
181. Morhart, P. *et al.* Maternal SARS-CoV-2 infection during pregnancy: possible impact on the infant. *European Journal of Pediatrics* (2021) doi:10.1007/s00431-021-04221-w.
182. Hcini, N. *et al.* Maternal, fetal and neonatal outcomes of large series of SARS-CoV-2 positive pregnancies in peripartum period: A single-center prospective comparative study. *European Journal of Obstetrics and Gynecology and Reproductive Biology* **257**, 11–18 (2021).
183. Pham, A., Aronoff, D. M. & Thompson, J. L. Maternal COVID-19, vaccination safety in pregnancy, and evidence of protective immunity. *Journal of Allergy and Clinical Immunology* **148**, 728–731 (2021).
184. Zhao, Y. *et al.* Follow-up Study on the Outcomes of Recovered Pregnant Women with a History of COVID-19 in the First and Second Trimesters: A Case Series from China. *Maternal-Fetal Medicine* **3**, 24–32 (2021).
185. Ayed, A. *et al.* Maternal and perinatal characteristics and outcomes of pregnancies complicated with COVID-19 in Kuwait. *BMC Pregnancy and Childbirth* **20**, 1–9 (2020).

186. Marton, T., Hargitai, B., Hunter, K., Pugh, M. & Murray, P. Massive Perivillous Fibrin Deposition and Chronic Histiocytic Intervillositis a Complication of SARS-CoV-2 Infection. *Pediatric and Developmental Pathology* **24**, 450–454 (2021).
187. Rabiei, M. *et al.* Maternal and fetal effects of COVID-19 virus on a complicated triplet pregnancy: a case report. *Journal of Medical Case Reports* **15**, (2021).
188. Hu, X. *et al.* Managing Preterm Infants Born to COVID-19 Mothers: Evidence from a Retrospective Cohort Study in Wuhan, China. *Neonatology* **117**, 592–598 (2021).
189. Lamba, V., Lien, J., Desai, J. & Talati, A. J. Management and short-term outcomes of neonates born to mothers with active perinatal SARS-CoV-2 infection. *BMC Pediatrics* **21**, (2021).
190. Vila-Candel, R. *et al.* Management of labour, puerperium, and lactation in SARS-CoV-2 positive women. Multicentric study in the Valencian Community. *Enfermeria Clinica* **31**, 184–188 (2021).
191. Halici-Ozturk, F. *et al.* Investigating the risk of maternal-fetal transmission of SARS-CoV-2 in early pregnancy. *Placenta* **106**, 25–29 (2021).
192. Sisman, J. *et al.* Intrauterine Transmission of SARS-COV-2 Infection in a Preterm Infant. *Pediatric Infectious Disease Journal* **39**, e265–e267 (2020).
193. Zaigham, M. *et al.* Intrauterine vertical SARS-CoV-2 infection: a case confirming transplacental transmission followed by divergence of the viral genome. *BJOG: An International Journal of Obstetrics and Gynaecology* **128**, 1388–1394 (2021).
194. Dong, L. *et al.* Evaluation of vertical transmission of SARS-CoV-2 in utero: Nine pregnant women and their newborns. *Placenta* **111**, 91–96 (2021).
195. von Kohorn, I. *et al.* In Utero Severe Acute Respiratory Syndrome Coronavirus 2 Infection. *Journal of the Pediatric Infectious Diseases Society* **9**, 769–771 (2020).
196. Giordano, G. *et al.* COVID-19 in pregnancy: placental pathological patterns and effect on perinatal outcome in five cases. *Diagnostic Pathology* **16**, (2021).
197. Levitan, D. *et al.* Histologic and Immunohistochemical Evaluation of 65 Placentas from Women with Polymerase Chain Reaction–Proven Severe Acute Respiratory Syndrome Coronavirus 2 (SARS-CoV-2) Infection. *Archives of Pathology and Laboratory Medicine* **145**, 648–656 (2021).
198. Nayak, A. H. *et al.* Impact of the Coronavirus Infection in Pregnancy: A Preliminary Study of 141 Patients. *Journal of Obstetrics and Gynecology of India* **70**, 256–261 (2020).
199. Gulersen, M. *et al.* Histopathologic evaluation of placentas after diagnosis of maternal severe acute respiratory syndrome coronavirus 2 infection. *American Journal of Obstetrics and Gynecology MFM* **2**, (2020).
200. Garcia-Manau, P. *et al.* Fetal Transient Skin Edema in Two Pregnant Women With Coronavirus Disease 2019 (COVID-19). *Obstetrics and gynecology* **136**, 1016–1020 (2020).
201. Timircan, M. *et al.* Exploring pregnancy outcomes associated with sars-cov-2 infection. *Medicina* **57**, 796–806 (2021).
202. Solís-García, G. *et al.* Epidemiology, management and risk of SARS-CoV-2 transmission in a cohort of newborns born to mothers diagnosed with COVID-19 infection. *Anales de Pediatría* **94**, 173–178 (2021).
203. Sevilla-Montoya, R. *et al.* Evidence of possible SARS-CoV-2 vertical transmission according to World Health Organization criteria in asymptomatic pregnant women. *Ultrasound in Obstetrics & Gynecology* **58**, 900–908 (2021).
204. Sun, M. *et al.* Evidence of mother-to-newborn infection with COVID-19. *British Journal of Anaesthesia* **125**, e245–e247 (2020).
205. Ronchi, A. *et al.* Evaluation of Rooming-in Practice for Neonates Born to Mothers with Severe Acute Respiratory Syndrome Coronavirus 2 Infection in Italy. *JAMA Pediatrics* **175**, 260–266 (2021).
206. Jan, M., Bhat, W. M., Rashid, M. & Ahad, B. Elective Cesarean Section in Obstetric COVID-19 Patients under Spinal Anesthesia: A Prospective Study. *Anesthesia, essays and researches* **14**, 611–614 (2020).

207. Hijona Elósegui, J. J. *et al.* Does the maternal-fetal transmission of SARS-CoV-2 occur during pregnancy? *Revista Clínica Española* **221**, 93–96 (2021).
208. Valk, J. E., Chong, A. M., Uhlemann, A. C. & Debelenko, L. Detection of SARS-CoV-2 in placental but not fetal tissues in the second trimester. *Journal of Perinatology* **41**, 1184–1186 (2021).
209. Fox, N. S. & Melka, S. COVID-19 in Pregnant Women: Case Series from One Large New York City Obstetrical Practice. *American Journal of Perinatology* **37**, 1002–1004 (2020).
210. Januszewski, M. *et al.* COVID-19 in pregnancy—perinatal outcomes and vertical transmission preventative strategies, when considering more transmissible SARS-CoV-2 variants. *Journal of Clinical Medicine* **10**, (2021).
211. Mongula, J. E. *et al.* COVID-19 during pregnancy: non-reassuring fetal heart rate, placental pathology and coagulopathy. *Ultrasound in Obstetrics and Gynecology* **56**, 773–776 (2020).
212. Hayakawa, S., Komine-Aizawa, S. & Mor, G. G. Covid-19 pandemic and pregnancy. *Journal of Obstetrics and Gynaecology Research* **46**, 1958–1966 (2020).
213. Damar Çakırca, T. *et al.* COVID-19 infection in pregnancy: a single center experience with 75 cases. *Ginekologia Polska* (2021) doi:10.5603/gp.a2021.0118.
214. Syed, S. *et al.* COVID-19 and Pregnancy Outcome: An Experience in 'COVID-19 Management Designated' Tertiary Care Hospital, Rawalpindi, Pakistan. *Journal of Rawalpindi Medical College* **24**, 85–91 (2020).
215. Farsi, Z. *et al.* Coronavirus disease-2019 infection in neonates of an infected pregnant mother with triplets. *Iranian Journal of Neonatology* **11**, 120–122 (2020).
216. Cosma, S. *et al.* Coronavirus disease 2019 and first-trimester spontaneous abortion: a case-control study of 225 pregnant patients. *American Journal of Obstetrics and Gynecology* **224**, 391.e1-391.e7 (2021).
217. Garcia-Ruiz, I. *et al.* Congenital infection of SARS-CoV-2 in live-born neonates: a population-based descriptive study. *Clinical Microbiology and Infection* **27**, 1521.e1-1521.e5 (2021).
218. Conti, M. G. *et al.* Consequences of early separation of maternal-newborn dyad in neonates born to SARS-CoV-2 positive mothers: An observational study. *International Journal of Environmental Research and Public Health* **18**, (2021).
219. Donati, S. *et al.* Coronavirus and birth in Italy: Results of a national population-based cohort study. *Annali dell'Istituto Superiore di Sanita* **56**, 378–389 (2020).
220. Ronnje, L. *et al.* Complicated COVID-19 in pregnancy: A case report with severe liver and coagulation dysfunction promptly improved by delivery. *BMC Pregnancy and Childbirth* **20**, (2020).
221. Kalamdani, P., Kalathingal, T., Manerkar, S. & Mondkar, J. Clinical Profile of SARS-CoV-2 Infected Neonates From a Tertiary Government Hospital in Mumbai, India. *Indian Pediatrics* **57**, 1143–1146 (2020).
222. Bachani, S. *et al.* Clinical Profile, Viral Load, Maternal-Fetal Outcomes of Pregnancy With COVID-19: 4-Week Retrospective, Tertiary Care Single-Centre Descriptive Study. *Journal of Obstetrics and Gynaecology Canada* **43**, 474–482 (2021).
223. Xu, S. *et al.* Clinical manifestation and neonatal outcomes of pregnant patients with coronavirus disease 2019 pneumonia in Wuhan, China. *Open Forum Infectious Diseases* **7**, (2020).
224. Pierce-Williams, R. A. M. *et al.* Clinical course of severe and critical coronavirus disease 2019 in hospitalized pregnancies: a United States cohort study. *American Journal of Obstetrics and Gynecology MFM* **2**, (2020).
225. Vega-Fernández, A. G. *et al.* Clinical and epidemiological characteristics of mothers with COVID-19 and their neonates: vertical transmission. *Medwave* **21**, e8454 (2021).
226. AlQurashi, M. A. *et al.* Clinical Characteristics of Newborn Infants Delivered to Pregnant Women With Laboratory-Confirmed COVID-19: A Single-Center Experience From Saudi Arabia. *Cureus* (2021) doi:10.7759/cureus.18573.

227. Liu, Y. *et al.* Clinical characteristics and outcome of SARS-CoV-2 infection during pregnancy. *Journal of Infection* **82**, e9–e10 (2021).
228. Liu, W. *et al.* Clinical Analysis of Neonates Born to Mothers with or without COVID-19: A Retrospective Analysis of 48 Cases from Two Neonatal Intensive Care Units in Hubei Province. *American Journal of Perinatology* **37**, 1317–1323 (2020).
229. Javad Nazari1 *et al.* Case-report of a neonate with COVID-19 infection: How he has been infected? *Iranian Journal of Neonatology* **12**, 104–109 (2021).
230. Vaezi, M., Mirghafourvand, M. & Hemmatzadeh, S. Characteristics, clinical and laboratory data and outcomes of pregnant women with confirmed SARS-CoV-2 infection admitted to Al-Zahra tertiary referral maternity center in Iran: a case series of 24 patients. *BMC Pregnancy and Childbirth* **21**, (2021).
231. Farghaly, M. A. A., Kupferman, F., Castillo, F. & Kim, R. M. Characteristics of Newborns Born to SARS-CoV-2-Positive Mothers: A Retrospective Cohort Study. *American Journal of Perinatology* **37**, 1310–1316 (2020).
232. Lu, D. *et al.* Asymptomatic COVID-19 infection in late pregnancy indicated no vertical transmission. *Journal of Medical Virology* **92**, 1660–1664 (2020).
233. Guo, Y. *et al.* Case series of 20 pregnant women with 2019 novel coronavirus disease in Wuhan, China. *Journal of Obstetrics and Gynaecology Research* **47**, 1344–1352 (2021).
234. Hascoët, J. M. *et al.* Case Series of COVID-19 Asymptomatic Newborns With Possible Intrapartum Transmission of SARS-CoV-2. *Frontiers in Pediatrics* **8**, (2020).
235. Kunjumon, B. *et al.* Breast Milk and Breastfeeding of Infants Born to SARS-CoV-2 Positive Mothers: A Prospective Observational Cohort Study. *American Journal of Perinatology* **38**, 1209–1216 (2021).
236. Woodworth, K. R. *et al.* Birth and Infant Outcomes Following Laboratory-Confirmed SARS-CoV-2 Infection in Pregnancy — SET-NET, 16 Jurisdictions, March 29–October 14, 2020. *MMWR* **69**, 1635–1641 (2020).
237. Massarotti, C. *et al.* Asymptomatic SARS-CoV-2 infections in pregnant patients in an Italian city during the complete lockdown. *Journal of Medical Virology* **93**, 1758–1760 (2021).
238. Citu, C. *et al.* Assessing SARS-CoV-2 Vertical Transmission and Neonatal Complications. *Journal of Clinical Medicine* **10**, 5253 (2021).
239. Fenizia, C. *et al.* Analysis of SARS-CoV-2 vertical transmission during pregnancy. *Nature Communications* **11**, (2020).
240. Oncel, M. Y. *et al.* A multicenter study on epidemiological and clinical characteristics of 125 newborns born to women infected with COVID-19 by Turkish Neonatal Society. *European Journal of Pediatrics* **180**, 733–742 (2021).
241. Nanavati, R., Mascarenhas, D., Goyal, M., Haribalakrishna, A. & Nataraj, G. A single-center observational study on clinical features and outcomes of 21 SARS-CoV-2-infected neonates from India. *European Journal of Pediatrics* **180**, 1895–1906 (2021).
242. Selvi, İ., Cevhar, F. & Biri, A. A Retrospective Analysis of Clinical Features, Maternal and Neonatal Outcomes of COVID-19 patients in an Obstetric Clinic in Ankara, Turkey. *Journal of Clinical Obstetrics & Gynecology* **31**, 72–76 (2021).
243. Ergon, E. *et al.* A novel case of neonatal acute respiratory distress syndrome with SARS-CoV-2 infection: potential perinatal transmission. *Archivos Argentinos de Pediatria* **119**, (2021).
244. Milbak, J. *et al.* A prospective cohort study of confirmed severe acute respiratory syndrome coronavirus 2 (SARS-CoV-2) infection during pregnancy evaluating SARS-CoV-2 antibodies in maternal and umbilical cord blood and SARS-CoV-2 in vaginal swabs. *Acta Obstetricia et Gynecologica Scandinavica* **100**, 2268–2277 (2021).
245. Hinojosa-Velasco, A. *et al.* A case report of newborn infant with severe COVID-19 in Mexico: Detection of SARS-CoV-2 in human breast milk and stool. *International Journal of Infectious Diseases* **100**, 21–24 (2020).

246. Bordbar, A., Kashaki, M., Rezaei, F. & Jafari, R. Vertical transmission of COVID-19 in a 1-day-old neonate. *Travel Medicine and Infectious Disease* **38**, (2020).
